# Optimizing enteral nutrition delivery by implementing volume-based feeding protocol for critically ill patients: an updated meta-analysis and systematic review

**DOI:** 10.1186/s13054-023-04439-0

**Published:** 2023-05-05

**Authors:** Lu Wang, Yu Wang, Hua-Xin Li, Rui-peng Zhang, Li Chang, Jun Zeng, Hua Jiang

**Affiliations:** 1 Institute for Emergency and Disaster Medicine, Sichuan Provincial People’s Hospital, University of Electronic Science and Technology of China, Chengdu, 610072 China; 2 Sichuan Provincial Research Center for Emergency Medicine and Critical Illness, Sichuan Provincial People’s Hospital, University of Electronic Science and Technology of China, Chengdu, 610072 China; 3 Department of Emergency Intensive Care Unit, Sichuan Provincial People’s Hospital, University of Electronic Science and Technology of China, Chengdu, 610072 Sichuan Province China

**Keywords:** Intensive care unit, Enteral nutrition, Volume-based feeding, Rate-based feeding

## Abstract

**Background:**

This study aims to provide an updated assessment of the efficacy of optimized enteral nutrition (EN) delivery by implementing the volume-based feeding (VBF) protocol in critically ill patients.

**Methods:**

We updated our previous literature retrieval with no language restrictions. The inclusion criteria were:1) Participants: Critically ill patients (Patients who was admitted in ICU; 2) Intervention: The VBF protocol was adopted for EN administration; 3) Comparison: The rate-based feeding (RBF) protocol was adopted for EN administration; 4) Major outcomes: EN nutrition delivery. The exclusion criteria included participants aged < 18 years, duplicated literature, animal and cellular experiments, and studies lacking any of the outcomes mentioned in the inclusion criteria. The databases included MEDLINE (through PubMed), Web of Science, Cochrane Library, Chinese Biomedical Literature Service System (SinoMed), Wanfang Data Knowledge Service Platform, and China National Knowledge Infrastructure.

**Result:**

Sixteen studies involving a total of 2896 critically ill patients are included in the updated meta-analysis. Compared with the previous meta-analysis, nine new studies were added that included 2205 more patients. The VBF protocol significantly improved energy (MD = 15.41%, 95% CI: [10.68, 20.14], *p* < 0.00001) and protein (MD = 22.05%, 95% CI: [10.89, 33.22], *p* = 0.0001) delivery. The patients in the VBF group stayed in the ICU for a shorter time (MD = 0.78, 95% CI: [0.01, 1.56], *p* = 0.05). The VBF protocol did not increase the risk of death (RR = 1.03, 95% CI: [0.85, 1.24], *p* = 0.76) or prolong the mechanical ventilation duration (MD = 0.81, 95% CI: [-0.30,1.92], *p* = 0.15). In addition, the VBF protocol did not affect EN complications, such as diarrhea (RR = 0.91, 95% CI: [0.73, 1.15], *p* = 0.43), emesis (RR = 1.23, 95% CI: [0.76, 1.99], *p* = 0.41), feeding intolerance (RR = 1.14, 95% CI: [0.63, 2.09], *p* = 0.66), and gastric retention (RR = 0.45, 95% CI: [0.16, 1.30], *p* = 0.14).

**Conclusion:**

Our study revealed that the VBF protocol significantly improved calorie and protein delivery in critically ill patients with no additional risk.

**Supplementary Information:**

The online version contains supplementary material available at 10.1186/s13054-023-04439-0.

## Background

Since the late 1990s, clinical practitioners have focused on enteral nutritional (EN) support for critically ill patients. However, delivery efficiency has not received equal attention [1, 2]. Underfeeding is a major challenge during EN therapy in critically ill patients. Researchers have revealed that elective stoppage of procedures in EN is one of the most important factors preventing the delivery of feeding [3]. Recent studies found that almost 70% of patients experienced EN interruption (ENI) in the intensive care unit (ICU), and the proportion of energy delivery was reduced by 19% per day compared with those without ENI [4]. Clinical evidence has demonstrated that longer the ENI time, higher the caloric inadequacy in critically ill patients [5]. However, there remains a lack of international consensus on how to reduce nutritional inadequacy in critically ill patients. Heyland et al. (2010) established a volume-based feeding (VBF) protocol which provided a solution for the under-delivery caused by ENI [6, 7]. This protocol targets the total amount of nutrient solution actually ingested by the patient. When a ENI occurs and enteral nutrition needs to be initiated again, the infusion rate is adjusted according to the remaining time and the remaining feeding volume to compensate for the underfeeding caused by the feeding interruption. Recently, the VBF protocol has gradually gained widespread attention, and related studies have been conducted. In 2021, we published the first systematic review and meta-analysis that evaluated the efficacy of the VBF protocol, and the results showed that the VBF protocol significantly improved the success rate of EN in critically ill patients [8]. After this systematic review was published, several well-designed, large-scale studies emerged. Therefore, the results of our meta-analysis need be updated. Here, we conducted a new systematic review and meta-analysis to evaluate the efficacy of optimizing EN delivery by implementing the VBF protocol for critically ill patients.

## Materials and methods

### Protocol registration and reporting format

This systematic review and meta-analysis followed the recommendations of the Joanna Briggs Institute Reviewers’ Manual and the Preferred Reporting Items for Systematic Reviews and Meta-analyses Statement [9, 10]. The protocol of this systematic review was registered in the PROSPERO database (identification number: CRD42022366084) [11].

### Eligibility criteria

#### Inclusion criteria

We defined the following inclusion criteria based on the participants, intervention, comparison, outcomes, and study design, i.e., PICOS method:

1. Participants: Critically ill patients hospitalized in the ICU
2. Intervention: The VBF protocol adopted for EN administration
3. Comparison: The rate-based feeding (RBF) protocol was adopted for EN administration
4. Outcome: ① Major outcomes: EN nutrition delivery, ② Secondary outcome: overall mortality including 60-day mortality, 28-day mortality, 7-day mortality, hospital mortality, and ICU mortality (when multiple mortality endpoints were reported in a trial, we prioritized the data as described before), length of ICU stays, length of hospital stays, mechanical ventilation duration, and incidence of adverse reactions such as emesis, diarrhea, feeding intolerance, and gastric retention
5. Study design: Randomized controlled trial (RCT) and cohort studies

#### Exclusion criteria

1. Age < 18 years
2. Duplicated literature
3. Animal experiments
4. Cellular experiments
5. Studies lacking any of the outcomes mentioned above

### Data sources and search strategy

We updated our previous literature retrieval (date of search, November 30, 2022) with no language restrictions. The databases included MEDLINE (through PubMed), Web of Science, Cochrane Library, Chinese Biomedical Literature Service System (SinoMed), Wanfang Data Knowledge Service Platform, and China National Knowledge Infrastructure. Moreover, we also retrieved from the Chinese Clinical Trial Registry (www.chictr.org.cn) and Clinicaltrials.gov. The following search terms were used for all databases: critical care, intensive care units, critical illness, volume-based feeding, Enhanced Protein-Energy Provision via the Enteral Route Feeding, nutrition support, and enteral nutrition, which were cross-referenced to the outcome terms. The complete search strategy used for all databases is presented in Additional file 1: Table S1. We also reviewed the reference lists of the relevant articles [12, 13].

### Data extraction

The data from the updated literature were independently extracted by two authors (Lu Wang and Hua-Xin Li) and merged with the previous data by Wang Lu after cross-checking. In addition, the reference lists of relevant literature were searched manually to identify any additional studies. Disagreements between the two reviewers were resolved by consensus or arbitration by a third reviewer (Hua Jiang).

### Assessment of study quality

We applied three different strategies to evaluate the quality of the literature, and this study was accomplished by two researchers (Lu Wang and Hua-Xin Li). The modified Jadad Scores Scale and revised Cochrane risk-of-bias tool for randomized trials were applied to RCTs, while the Newcastle–Ottawa Scale (NOS) was used for cohort studies [14–16]. A high-quality study was defined as an RCT that scored > 3 on the modified Jadad scale or a cohort study with a score of > 5 on the NOS. The strength of evidence for each outcome was evaluated using the GRADEpro online website tool, which is used to create Summary of Findings table [17].

### Statistical method

RevMan 5.4 was chosen as the meta-analysis tool for this study. For continuous variables, standardized mean difference or weighted mean difference deviations were used. For dichotomous variables, 95% confidence intervals (CI) consistent with the rate ratios (RR) were used.

### Heterogeneity analysis

We assessed the heterogeneity of the combined data using the following steps: First, we used an I^2^ measure to assess whether there was any heterogeneity between the combined literature. I^2^ ≥ 75% showed high heterogeneity, 50% ≤ I^2^ < 75% showed moderate heterogeneity, and 25 ≤ I^2^ < 50% showed low heterogeneity. If I^2^ = 0, we used the fixed-effect model for data analysis; otherwise, the random-effect model was used [18]. If there was any heterogeneity among the combined data, we conducted a sensitivity analysis or subgroup analysis to analyze the source of heterogeneity.

## Results

### The results of literature retrieval

**Fig. 1:**
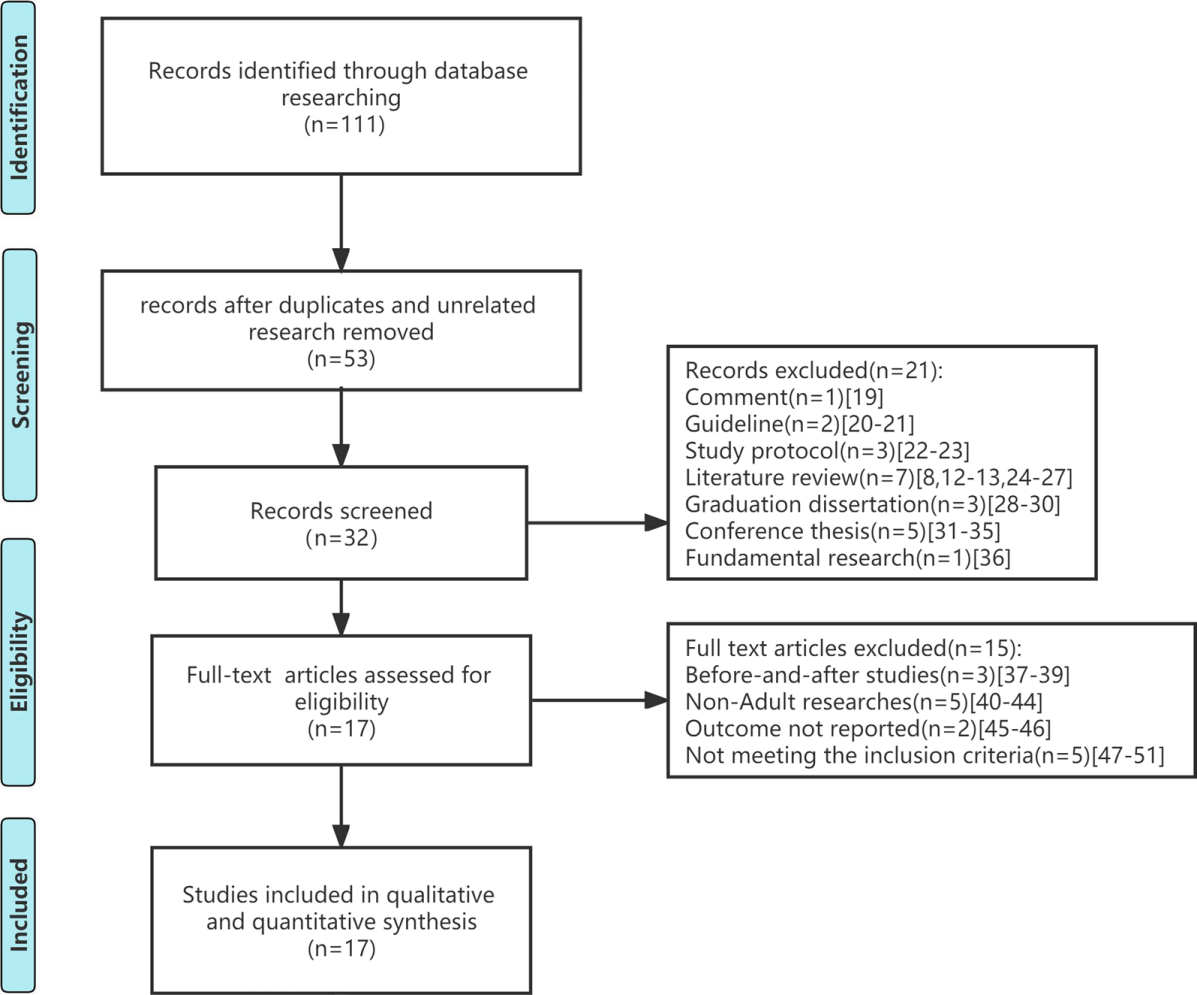
The literature research process

The literature search process is illustrated in Fig. 1. Following the removal of duplicates and unrelated studies, 53 records were screened based on their titles and abstract [8, 12, 13, 19–68]. The full-text assessment was performed on 17 articles. According to the inclusion criteria above, there were 17 studies, including qualitative synthesis and quantitative synthesis. According to the results of the quality assessment shown in Additional files 2-4: Tables s2-s4), only 1 study was assessed as being of low quality among all enrolled studies, and 8 studies were of high quality.

### Data extraction result

A unified data extraction table was developed to extract the characteristics of the included studies. The characteristics of the included trials are presented in Tables 1, 2. Among the 17 studies, the majority reported the proportion of calorie delivery (16 studies), ICU length of stay (10 studies), mechanical ventilation duration (9 studies), and mortality (10 studies). Compared with the previous study, we found that more recent studies reported the incidence of complications such as diarrhea, emesis, and gastric retention.

**Table 1:** Details of data extraction (Part 1)

Author, year	Study type	P.T	N.O.P (ITT)	Proportion of calorie delivery (%)		Completion of 80% energy delivery (n/N)		Proportion of protein delivery (%)		Patients with feeding intolerance (n/N)	
				VBF	RBF	VBF	RBF	VBF	RBF	VBF	RBF
Daren K Heyland [59]	RCT	ICU patients with mechanical ventilation	519 (252 vs. 267)	43.6 + 32.1	33.6 + 29.5	NM	NM	47.4 + 34.7	33.8 + 29.9	NM	NM
Stephen A. McClave [64]	RCT	Critical patients	57 (37 vs. 20)	92.9 ± 16.8	80.9 ± 18.9	NM	NM	NM	NM	NM	NM
Kate Fetterplace [57]	RCT	Critical patients	60 (30 vs. 30)	84 ± 21	73 ± 11	NM	NM	90 ± 25	57 ± 8	9/30	8/30
Yanxia Lu [52]	RCT	Critical patients	56 (28 vs. 28)	92 ± 80	84 ± 10	NM	NM	NM	NM	NM	NM
Guiyan Qi [53]	RCT	Critical patients with ventilation	120 (60 vs. 60)	77.4 ± 13.8	53.6 ± 13.3	NM	NM	NM	NM	27/60	23/60
Shuangshuang Yang [54]	RCT	ICU patients with mechanical ventilation	121 (61 vs. 60)	93 ± 24	82 ± 18	NM	NM	NM	NM	11/61	7/60
Ivy N. Haskins [58]	Cohort study	Critical patients	77 (39 vs. 38)	74.01	57.02	NM	NM	NM	NM	NM	NM
Susan Roberts [66]	Cohort study	Critical patients	171 (56 vs. 53)	79.6 ± 14.5	67.6 ± 17.8	NM	NM	79.3 ± 13.7	68.6 ± 16.4	NM	NM
Elizabeth D. Krebs [62]	Cohort study	Trauma, burn and surgical critical patients	99 (50 vs. 49)	84.5 (67.5–91.9)	73.4 (58.6–83.6)	NM	NM	86.2 (72.1–93.7)	77.4 (61.0–87.8)	9/50	15/49
Gaurav Sachdev [67]	Cohort study	Trauma critical patients	222 (78 vs. 144)	73.3 ± 13.3	65 ± 15.3	25/78	24/144	NM	NM	NM	NM
JaNae Kinikin [61]	Cohort study	Critical patients	70 (35 vs. 35)	80.9 ± 13.5	66.5 ± 24.0	NM	NM	NM	NM	NM	NM
Amanda Holyk [60]	Cohort study	Critical patients	189 (89 vs. 100)	102.00	75	63/89	42/100	87	68	NM	NM
Mina Bharal [55]	Cohort study	ICU patients with mechanical ventilation	82 (55 vs. 27)	77.8 ± 13.4	46.1 ± 19.7	NM	NM	72.9 ± 15.0	40.1 ± 18.9	5/55	7/27
Travis Swiatlo [68]	Cohort study	Trauma, and surgical critical patients	283 (77 vs. 206)	93.1 ± 11.3	71.3 ± 35.8	NM	NM	NM	NM	NM	NM
Phillip J. Prest [65]	Cohort study	Trauma, and surgical critical patients	492 (295 vs. 197)	NM	NM	1726/3028	678/2523	NM	NM	NM	NM
Angela Bonomo [56]	Cohort study	Surgical critical patients	73 (63 vs. 10)	99.8 (69–108)	67.5 (62–75)	NM	NM	NM	NM	NM	NM
Jason McCartt [63]	Cohort study	Trauma, and surgical critical patients	488 (232 vs. 256)	85.50	75.30	167/232	122/256	NM	NM	52/2059	29/2663

**Table 2:** Details of data extraction (Part 2)

Author, year	Emesis (n/N)		Diarrhea (n/N)		Gastric retention (n/N)		ICU length of stay (d)		Mechanical ventilation duration (d)		Mortality (n/N)	
	VBF	RBF	VBF	RBF	VBF	RBF	VBF	RBF	VBF	RBF	VBF	RBF
Daren K Heyland [59]	NM	NM	NM	NM	NM	NM	7.2 (3.4–11.1)	5.7 (2.8–11.8)	4.3 (1.3–9.9)	3.0 (1.4–7.3)	68/252	63/267
Stephen A. McClave [64]	NM	NM	NM	NM	NM	NM	NM	NM	NM	NM	NM	NM
Kate Fetterplace [57]	NM	NM	16/30	16/30	NM	NM	10.6 ± 8.3	9.1 ± 5.5	8.7 ± 7.5	7.0 ± 5.0	4/30	5/30
Yanxia Lu [52]	6/28	8/28	13/28	10/28	NM	NM	NM	NM	NM	NM	NM	NM
Guiyan Qi [53]	NM	NM	NM	NM	NM	NM	8.1 ± 2.2	9.0 ± 2.8	6.6 ± 2.2	7.9 ± 2.3	4/60	6/60
Shuangshuang Yang [54]	12/61	9/60	17/61	14/60	NM	NM	NM	NM	NM	NM	NM	NM
Ivy N. Haskins [58]	NM	NM	NM	NM	NM	NM	14 (10–21)	9 (5–19)	9 (7–16)	5 (3–12)	4/39	5/38
Susan Roberts [66]	NM	NM	NM	NM	NM	NM	13.8 ± 9.7	14.1 ± 12.9	11.4 ± 9.5	11.0 ± 11.4	NM	NM
Elizabeth D. Krebs [62]	6/50	7/49	32/50	26/49	4/50	1/49	14 (10.0–23.0)	15 (11.0–22.0)	NM	NM	7/50	7/49
Gaurav Sachdev [67]	1/78	2/144	4/78	6/144	NM	NM	13 ± 6.2	14 ± 7.6	13 ± 7.7	14 ± 11.3	10/78	25/144
JaNae Kinikin [61]	NM	NM	NM	NM	NM	NM	NM	NM	NM	NM	NM	NM
Amanda Holyk [60]	NM	NM	NM	NM	3 /89	2/100	5 (3–9)	5.7 (3.8–10.7)	7 (4–12)	6.6 (2.5–18.8)	12/89	12/100
Mina Bharal [55]	NM	NM	NM	NM	7/55	2/27	11 (7–19)	10 (6–15)	9 (6–15)	6 (4–10)	12/55	6/27
Travis Swiatlo [68]	1/77	13/206	9/77	31/206	NM	NM	NM	NM	NM	NM	26/77	68/206
Phillip J. Prest [65]	NM	NM	NM	NM	NM	NM	NM	NM	NM	NM	NM	NM
Angela Bonomo [56]	NM	NM	NM	NM	NM	NM	10 (3–34)	10 (3–23)	5 (0–30)	6.5 (0–23)	17/63	5/10
Jason McCartt [63]	NM	NM	NM	NM	NM	NM	NM	NM	NM	NM	NM	NM

NM: not mentioned.

## Results of meta-analysis

### The efficacy of EN delivery

#### Energy delivery

As shown in Fig. 3a, 7 studies reported daily actual calorie intake, involving 1019 patients in total. There was high heterogeneity in daily actual calorie intake (I^2^ = 97%). The results indicated that the daily actual calorie intake was significantly higher in the VBF group than the RBF group (mean difference [MD] = 386.61 kcal/d, 95% CI: [180.32, 592.91], *p* = 0.0002); thus, we selected the proportion of calorie delivery and completion of 80% energy delivery to evaluate the efficacy of energy delivery of EN treatment in the ICU.

#### Proportion of calorie delivery

Compared with the prior study, 11 studies reported the proportion of calorie delivery involving 1699 patients, and 6 of them were new studies. As shown in Fig. 3b, there was high heterogeneity among the enrolled studies (I^2^ = 81%). However, the size effect was higher on improvement in the VBF group for the efficacy of energy delivery in the random-effect model (MD = 15.41%, 95% CI: [10.68, 20.14], *p* < 0.00001).

**Fig. 2:**
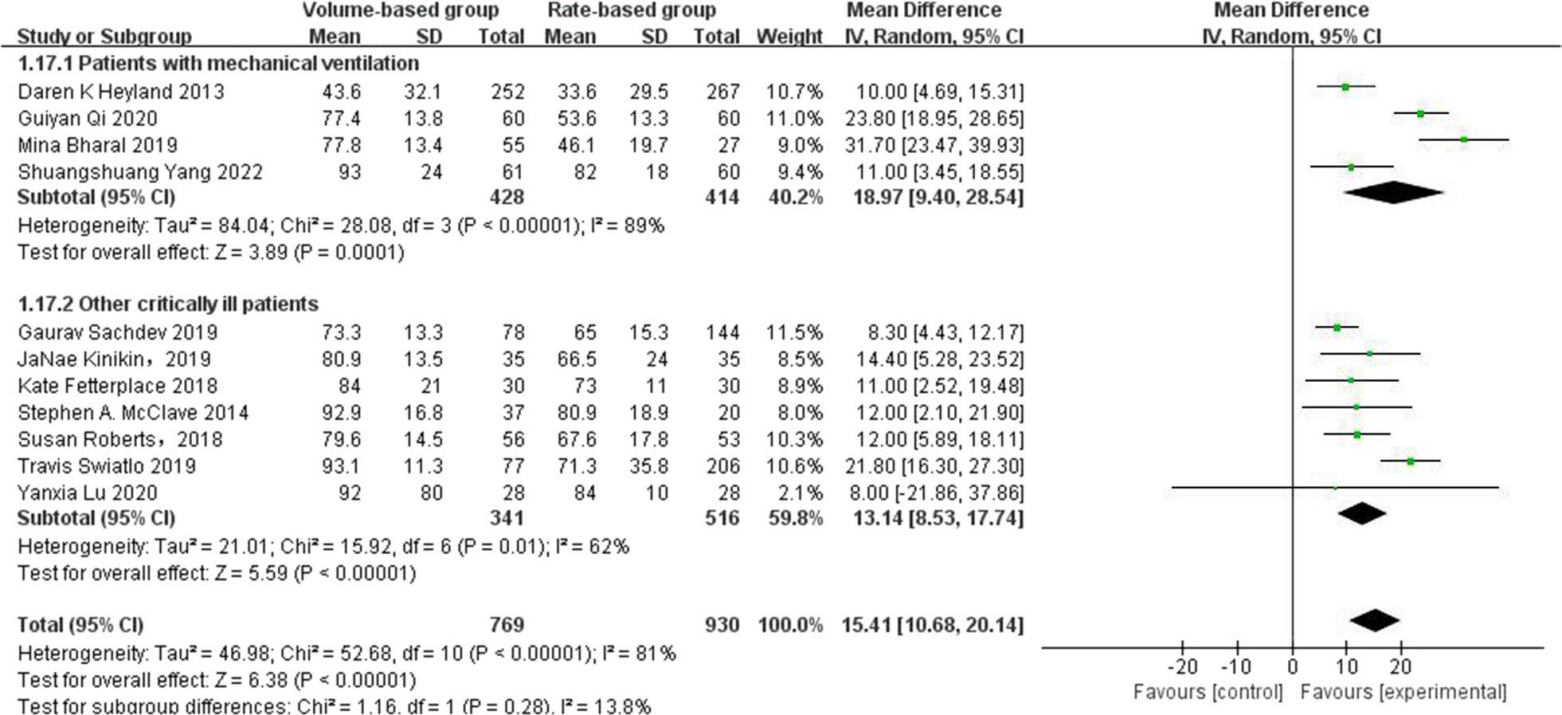
Forest plot of meta-analysis of proportion of calorie delivery in two subgroups, including critical patients and trauma critical patients and the patients with mechanical ventilation

We also performed a subgroup analysis. We compared the proportions of calorie delivery between patients who received mechanical ventilation and those who were critically ill. As shown in (Fig. 2), there was no significant difference between the two subgroups [*p* = 0.28, I^2^ = 13.8%].

#### Completion of 80% energy delivery

**Fig. 3:**
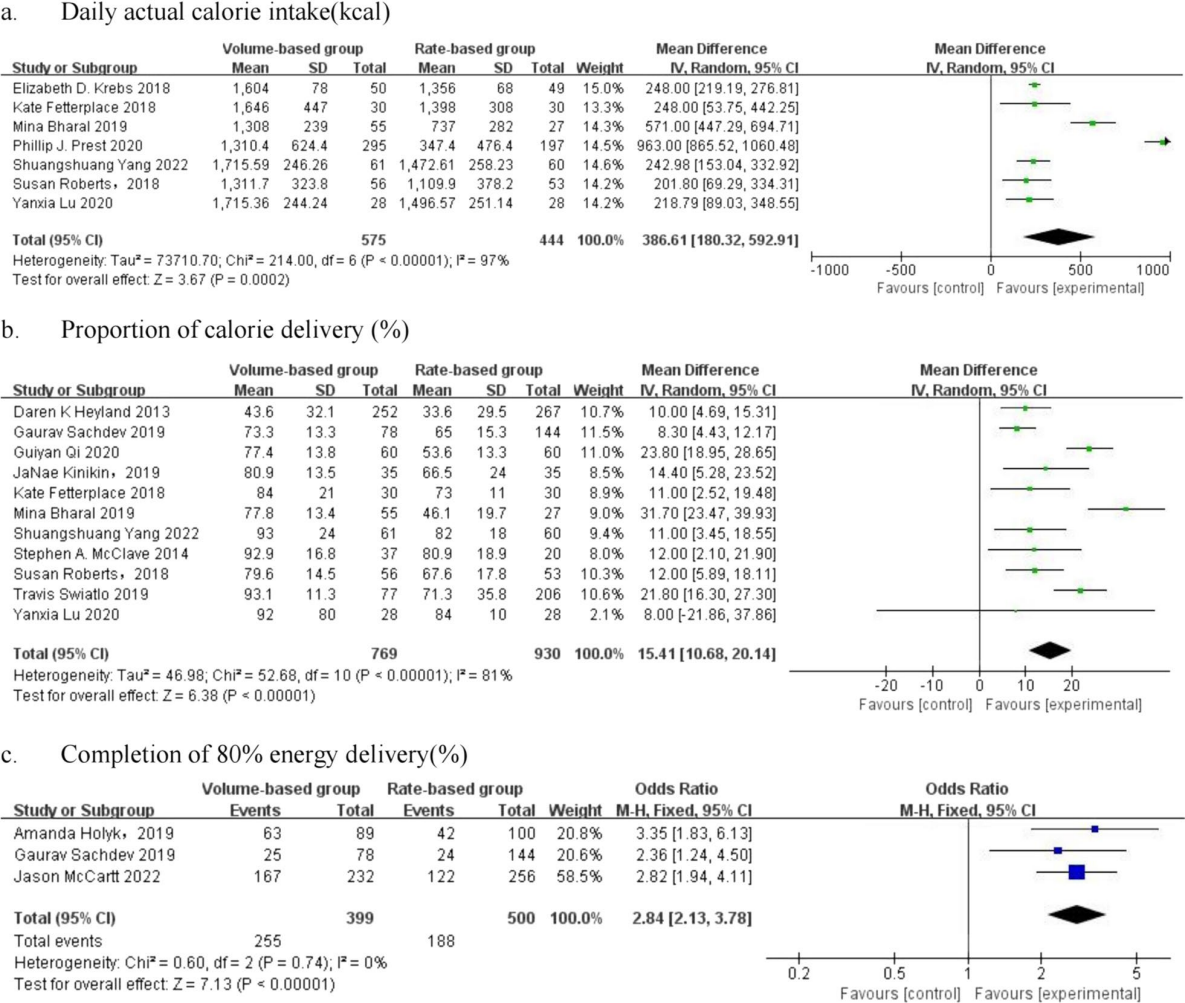
Forest plot of meta-analysis of energy delivery in the two groups

This was one additional assessment metric in this updated systematic review compared with the previous one of 2021. One study by Prest et al. also reported these metrics; however, they calculated the target days based on the total number of hospitalized days for all patients rather than the target population in the total population. Therefore, this study was excluded. Three studies involved 899 samples, 500 of which received the VBF protocol. We used a fixed-effect model because there was no heterogeneity between the enrolled studies (I^2^ = 0%), and the VBF protocol achieved significantly higher completion of energy delivery for critically ill patients (OR = 2.84, 95% CI: [2.13, 3.78], *p* < 0.00001) (Fig. 3c).

#### Protein delivery

**Fig. 4:**
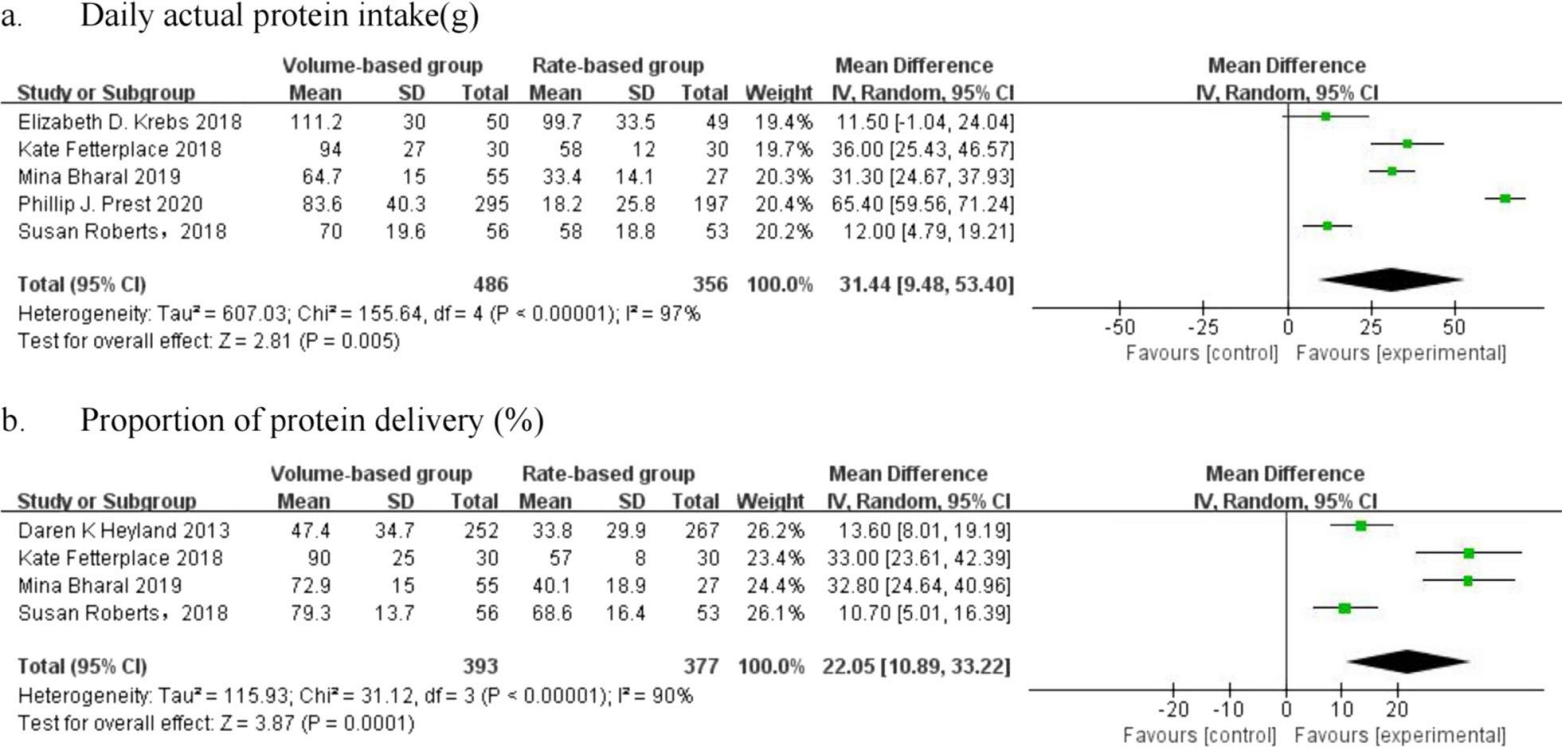
Forest plot of meta-analysis of protein delivery in the two groups

Two metrics were reported to measure protein delivery: daily actual protein intake and proportion of protein delivery. Three of the six studies reported both metrics. In summary, 842 patients were included in the analysis of daily actual protein intake, and 770 patients were included in the analysis of the proportion of protein delivery. There was high heterogeneity among the enrolled studies. The daily actual protein intake (MD = 31.44 g/d, 95% CI: [9.48, 53.4], *p* = 0.005, I^2^ = 97%) and the proportion of protein delivery (MD = 22.05%, 95% CI: [10.89, 33.22], *p* = 0.0001, I^2^ = 90%) in the VBF group were significantly higher than those in the RBF group (Fig. 4).

### Outcomes

#### Mortality

**Fig. 5:**
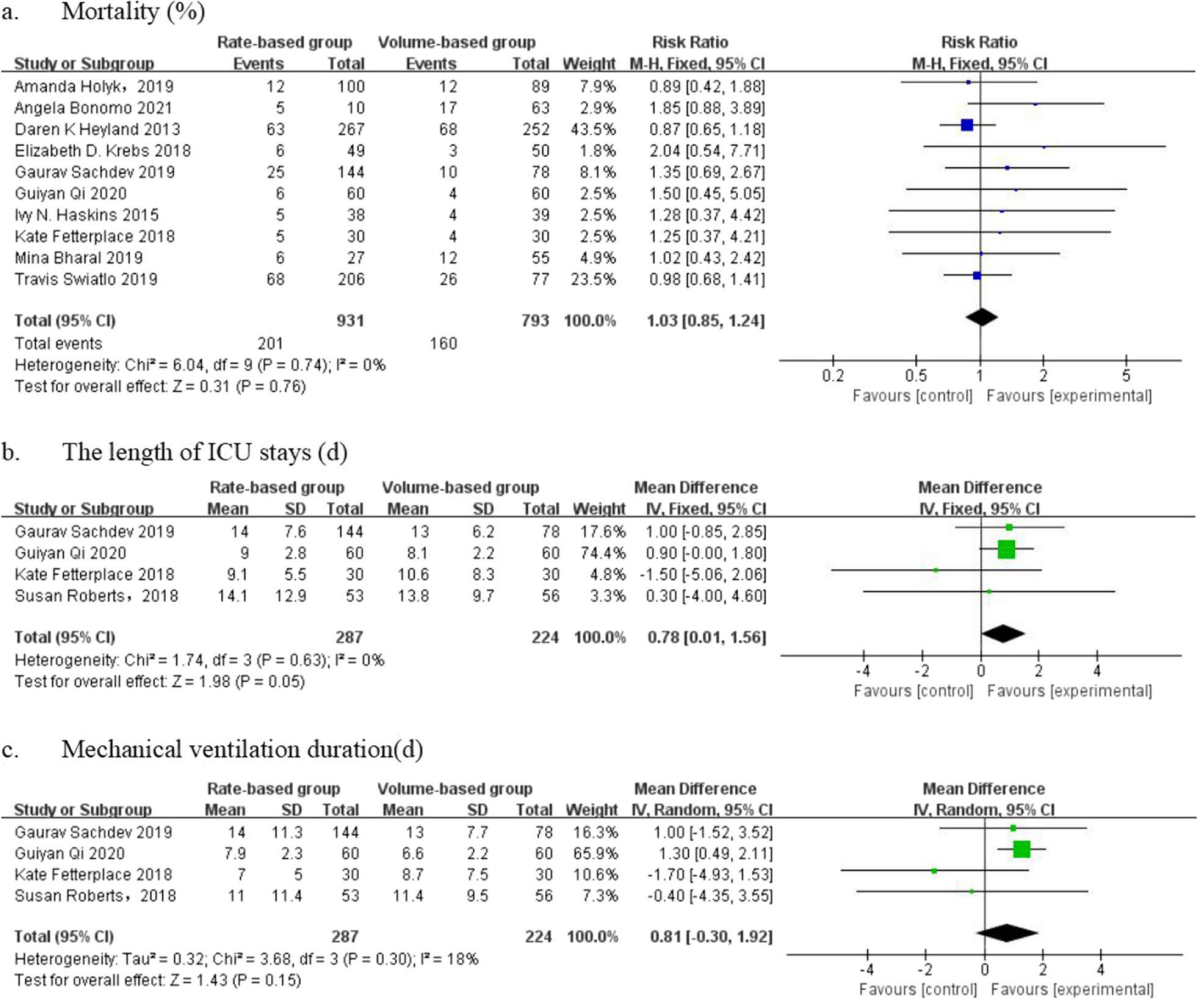
Forest plot of meta-analysis of outcomes in the two groups

Nine studies involving 1441 patients reported mortality. Compared with the previous study, four studies with 863 patients were included. According to the analysis, there was no heterogeneity between the enrolled studies; therefore, the fixed-effect model was applied. The pooled results indicated that the mortality rate of the VBF group was not significantly different from that of the RBF group (rate ratio [RR] = 1.03, 95% CI: [0.85, 1.24], *p* = 0.76) (Fig. 5a).

#### The length of ICU stays

Four studies (one new study) reported the length of ICU stays, involving 511 patients, 224 of whom belonged to the VBF group. We used a fixed-effect model in the meta-analysis of the length of ICU stays (I^2^ = 0%). According to the results, the length of ICU stays in the VBF group was significantly reduced compared with the RBF group (MD = 0.78, 95% CI: [0.01, 1.56], *p* = 0.05) (Fig. 5b).

#### Mechanical ventilation duration

We used a random-effect model for mechanical ventilation duration (I^2^ = 18%). In contrast to the previous study, we did not find a significant difference in mechanical ventilation duration between the VBF and RBF groups (MD = 0.81, 95% CI: [-0.30,1.92], *p* = 0.15) (Fig. 5c).

### Complications

#### Emesis and diarrhea

**Fig. 6:**
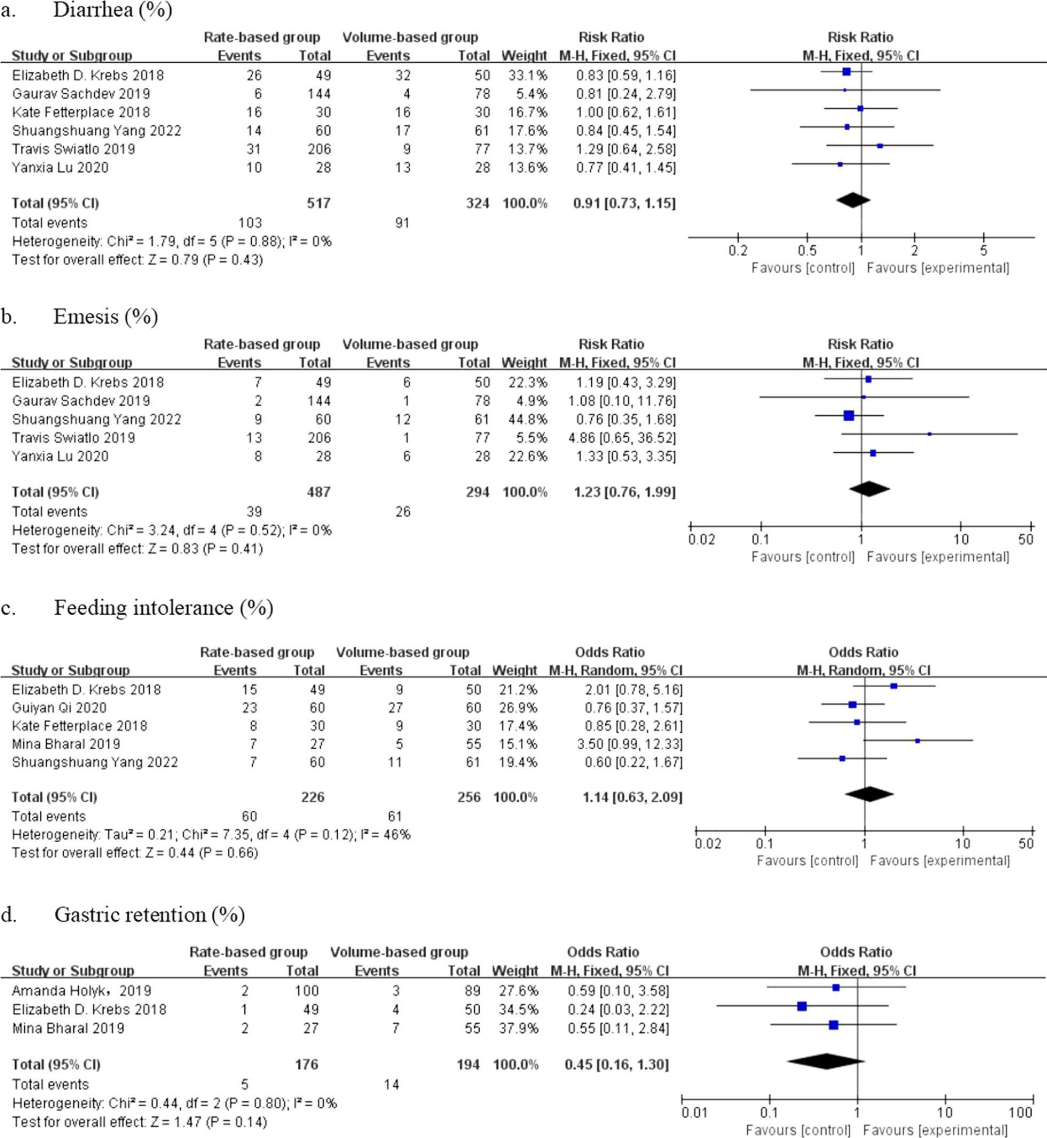
Forest plot of meta-analysis of adverse reactions in the two groups

Two recent studies reported the incidence of diarrhea and emesis in 486 patients. We used fixed-effect models because there were no heterogeneities in either analysis (I^2^ = 0%). According to the results, we found that the incidence of diarrhea (RR = 0.91, 95% CI: [0.73, 1.15], *p* = 0.43) and emesis (RR = 1.23, 95% CI: [0.76, 1.99], *p* = 0.41) in the VBF group did not increase significantly compared with that in the RBF group (Fig. 6a and b).

#### Feeding intolerance

Five studies reported the incidence of feeding intolerance involving 482 patients. According to the heterogeneity analysis, the random-effect model was used for the analysis of feeding intolerance (I^2^ = 46%). The results showed that the incidence of feeding intolerance in the VBF group was not significantly different from that in the RBF group (RR = 1.14, 95% CI: [0.63, 2.09], *p* = 0.66) (Fig. 6c).

#### Gastric retention

A recent study reported the incidence of gastric retention, which was the proportion of patients who reached the extreme boundary of gastric residual volume (GRV). Three studies involving 370 patients were included in the analysis. We used the fixed-effect model to analyze the data because the value of I^2^ was 0%. According to the results, the difference between the two groups was not significant (RR = 0.45, 95% CI: [0.16, 1.30], *p* = 0.14) (Fig. 6d).

#### Sensitivity analysis

For meta-analysis with heterogeneity (I^2^ ≠ 0), we conducted a sensitivity analysis to explore the source of heterogeneity. We divided the analysis into the following two parts: 1) comparing each study and determining the possible sources that may cause the heterogeneity of the meta-analysis; 2) excluding the related literature and observing the value of I^2^. If the value was reduced to zero, the heterogeneity was removed. Meanwhile, the excluded studies were identified as sources of heterogeneity.

#### Delivery of calorie and protein

Three studies reported the proportion of calorie delivery and did not report the daily actual calorie intake, including studies by Qi et al., Kinikin et al., and McClave et al. We realized that the value of the study by Qi et al. was significantly different from that of the others. Moreover, a study by Bharal et al. adjusted for Acute Physiology And Chronic Health Evaluation II(APACHE II) score, admission type, method of estimated energy requirement, and time to start EN. In addition, the differences in numbers between the two groups in the study by Swiatlo et al. were too large. Thus, we excluded the above studies, and the value of I^2^ was reduced from 81 to 0%. The proportion of calorie delivery in the VBF group was still significantly higher than that in the RBF group (MD = 10.23%, 95% CI: [7.9, 12.56], *p* < 0.00001) (Additional file 5: Figure S1a).

After screening the literature, we found that Prest et al. used a different procedure to calculate the average daily calorie and protein intake. This study calculated related metrics for EN delivery by considering the EN duration (days) of the included patients as the denominator, while the others considered the population (patient count). Moreover, as mentioned, the values in the study by Bharal et al. were adjusted for the APACHE II score, admission type, method of estimated energy requirement, and time to start EN. After excluding the above studies, there was no heterogeneity between the remaining studies (I^2^ = 0) on the outcome of daily calorie intake. However, there was still heterogeneity in the outcomes of daily actual protein intake. In the study by Fetterplace et al., we found that patients in the two groups received different doses of protein. Thus, we excluded the study from the meta-analysis of both daily actual protein intake and proportion of protein delivery, and the heterogeneity was diminished (I^2^ = 0). The results showed that the daily actual calorie intake (MD = 244.61 kcal/d, 95% CI: [218.54, 270.68], *p* < 0.00001), daily actual protein intake (MD = 11.88 g/d, 95% CI: [5.63, 18.12], *p* = 0.0002), and proportion of protein delivery (MD = 12.18%, 95% CI: [8.19, 16.16], *p* < 0.00001) in the VBF group were significantly higher than those in the RBF group (Additional file 5: Figures S1b, S1c, S1d).

#### Mechanical ventilation duration

Qi et al. enrolled only critical patients with ventilation, which may have affected the distribution of samples in this part of the analysis. Thus, we excluded this study, and the heterogeneity was diminished (I^2^ = 0). The results showed no significant difference between the two groups (MD = -0.10, 95% CI: [-1.87, 1.68], *p* = 0.91) (Additional file 5: Figure S1e).

#### Feeding intolerance

While screening the relevant literature, we found that the studies by Krebs et al. and Bharal et al. were both cohort studies, and the others were all RCTs. We excluded cohort studies, and there was no heterogeneity among the remaining studies (I^2^ = 0). The two groups showed no significant difference in terms of incidence of feeding intolerance (MD = 0.73, 95% CI: [0.43, 1.23], *p* = 0.24) (Additional file 5: Figure S1f).

## Discussion

Our previous systematic review enrolled only 7 studies involving 691 patients. In the current updated systematic review, study number and sample size were significantly increased (10 new studies enrolling 2488 patients). Moreover, many more types of patients (or clinical conditions) receiving VBF have been evaluated, such as trauma patients and patients with mechanical ventilation.

Sufficient delivery of energy and protein is crucial for critically ill patients receiving EN. Critically ill patients always face an extremely high risk of malnutrition, and severe malnutrition leads to worse clinical outcomes and even increases mortality [69, 70]. In addition, growing evidence suggests that the gastrointestinal tract plays an important role in maintaining balance during health and disease [71, 72]. For critically ill patients, EN is a cornerstone of therapy for maintaining gastrointestinal function and avoiding microbial translocation in the gastrointestinal tract [73]. Inadequate enteral feeding is a widespread problem in ICUs and can cause patients to be in a highly catabolic condition with inadequate nutritional support [74]. However, ENI is still underappreciated as one of the major causes of inadequate delivery of EN [75]. A recent study indicated that only 26% of ENI were deemed “avoidable” [76], and ENI always led to 11.5% of the average daily calorie deficit. However, more than 80% of interruptions lack clear written instructions [77]. Therefore, clinicians should pay more attention to the delivery of EN, especially the incidence of ENI in critically ill patients.

Our study revealed that the VBF protocol significantly improved calorie and protein delivery for critically ill patients with no additional risk, which also minimized the disturbance of ENI to EN delivery. Heyland et al. introduced the VBF protocol in 2010 and conducted several clinical trials to demonstrate its efficacy in improving energy and protein deliveries. [6, 38, 45]. Since then, many researchers have focused on this protocol and applied it to critically ill patients. In addition to the RCTs and cohort studies included in this meta-analysis, there are some before-and-after studies in critically ill patients and other studies in non-severely ill patients [27, 37, 39]. These studies found that the VBF protocol performs better than the RBF protocol. Notably, although some studies did not report the clinical outcomes of VBF, they applied VBF protocols as a method of quality control for EN management [48, 49]. They found that the VBF protocol is easy to implement for clinical staff and helpful in ensuring that the participants receive equal amounts of EN support in their studies. These studies provide further validation of the efficacy of the VBF protocol. Our study summarizes the current VBF research and proves that the VBF protocol is beneficial for patients with EN. According to the evaluation using the GRADEpro tool in Additional file 6: Table S5, the certainty of meta-analysis for EN delivery was significantly elevated compared with previous systematic reviews and meta-analyses. This current systematic review and meta-analysis provide clearer guidance to clinical practitioners.

Most published studies did not report the incidence of gastric retention, and we emphasized the importance of monitoring GRVs in our prior study. Although the extreme boundary of GRV varies in different enrolled studies, we did not find any evidence indicating that the VBF protocol would increase the GRV for critically ill patients. This conclusion confirms that the VBF protocol is safe for optimizing EN delivery. Several studies have questioned the efficacy of GRV in monitoring gastrointestinal function in critically ill patients [78]. A recent meta-analysis indicated that not monitoring GRV decreased the rate of feeding intolerance in critically ill patients and did not result in an increase in the mortality rate compared with monitoring of GRV [79]. For safety consideration, we still suggest that GRV is still an irreplaceable indicator in related studies to assess the more accurate value of daily actual intake of enteral nutrition for critically ill patients, although it does not reflect the gastrointestinal function very precisely..

Our study had several limitations. First, the EN formulation varied in different studies, and some of them did not mention the main formulation in their literature. Thus, it is difficult to measure the bias towards different EN protocols. And different studies follow different criteria in proportion of calorie and protein delivery for patients, which may also bias the results. In addition, the definition of complications, such as diarrhea, emesis, and feeding intolerance, was still not uniform in our study. Finally, it was regrettable that we were unable to conduct a meta-analysis of the infectious complications in our studies because there were inadequate study reporting the occurrence of infectious complications.

Compared with prior studies, ours provides more evidence to validate the efficacy of optimized delivery in implementing the VBF protocol for critically ill patients. In addition, we confirmed that the VBF protocol did not increase the risk of poor prognosis or complications.

## Conclusion

Our study provides strong evidence to validate the efficacy and safety of optimized delivery by implementing the VBF protocol and also provides an important rationale for the application of VBF protocols in clinical settings.

## Supplementary Information

**Additional file 1. Table S1.** Literature research strategy, databases, and key words.

**Additional file 2. Table S2.** The results of quality assessment on Jadad for RCTs.

**Additional file 3. Table S3.** The results of quality assessment on NOS for cohort studies.

**Additional file 4. Table S4.** The results of quality assessment on ROB2 for RCT.

**Additional file 5. Figure S1.** The results of sensitivity analysis.

**Additional file 6. Table S5.** The results of Grade of Recommendations Assessment, Development and Evaluation.

## Data Availability

All data related to the present systematic review and meta-analysis are available from the corresponding author on reasonable request.

## Abbreviations

VBF: Volume-based feeding;
EN: Enteral nutrition
ICU: Intensive care unit
ENI: Enteral nutrition interruption
RBF: Rate-based feeding
RCT: Randomized controlled trial
NOS: Newcastle–Ottawa Scale
P.T.: Patient type
N.O.P.: Number of patients
ITT: Intention-To-Treat
NM: Not mentioned
RR: Rate ratio
MD: Mean difference
GRV: Gastric residual volume
CI: Confidence interval
SD: Standard deviation
