# Supplementary material for: Optimizing enteral nutrition delivery by implementing volume-based feeding protocol for critically ill patients: an updated meta-analysis and systematic review
Source: Crit Care. 2023 May 5;27:173. doi: 10.1186/s13054-023-04439-0 (PMC10161662; doi:10.1186/s13054-023-04439-0)
Supplement: Supplementary file 1 — Additional file 1. Table S1. Literature research strategy, databases, and key words. [file 13054_2023_4439_MOESM1_ESM.docx]

| Databases | Strategy |
| --- | --- |
| PubMed | ((volume-based [Title/Abstract]) OR (Enhanced Protein-Energy Provision via the Enteral Route Feeding [Title/Abstract]) OR (PEP uP [Title/Abstract]) OR (Enhanced enteral nutrition [Title/Abstract]) OR (Improved Enteral nutrition [Title/Abstract])) AND (rate-based [Title/Abstract])) AND ((enteral nutrition [Title/Abstract]) OR ( nutrition [Title/Abstract])) AND ((critical [Title/Abstract]) OR (intensive care [Title/Abstract]) OR (sever patient [Title/Abstract])) |
| Web of Science | #1:TS=(volume-based)  #2:TS=(Enhanced Protein-Energy Provision via the Enteral Route Feeding)  #2:TS #3:TS= (PEP uP)  #4:TS= Rate-based  #5:TS= (enteral nutrition)  #6:TS= (EN)  #7:TS= (critical)  #8:TS= (intensive care)  #9: #1 OR #2 OR #3  #10: #5 OR #6  #11: #7 IR #8  #12: #9 AND #10 and #11  time span:2000.01.01-2022.11.30. Index: SCI-EXPANDED, SSCI, A&HCI, CPCI-S, CPCI-SSH, BKCI-S, BKCI-SSH, ESCI, CCR-EXPANDED, IC. |
| Cochrane Library | #1:TS= volume-based  #2:TS #2:TS=Enhanced Protein-Energy Provision via the Enteral Route Feeding  #3:TS= PEP uP  #4:TS= Rate-based  #5:TS= enteral nutrition  #6:TS= EN  #7:TS= critical  #8:TS= intensive care  #9: #1 OR #2 OR #3  #10: #5 OR #6  #11: #7 IR #8  #12: #9 AND #10 and #11 |
| SINOMED | ("Ji Yu Rong Liang"[Abstract: Intelligent] OR “Tong Guo Chang Dao Tu Jing Zeng Qiang Dan Bai Zhi-Neng Liang Gong Ying” [Abstract: Intelligent]) AND "Ji Yu Su Lv"[Abstract: Intelligent] AND "Chang Nei Ying Yang"[Abstract: Intelligent] AND "Zhong Zheng"[Abstract: Intelligent] |
| CNKI | Search Condition: (((Abstract = Ji Yu Rong Liang) OR (Abstract = Tong Guo Chang Dao Tu Jing Zeng Qiang Dan Bai Zhi-Neng Liang Gong Ying)) AND (Abstract = Ji Yu Su Lv) AND (Abstract = Chang Nei Ying Yang) AND (Abstract = Zhong Zheng)) and dateline between (2000-01-01,2022-11-30) |

**Table S1.** Literature research strategy, databases and key words
