# Supplementary material for: Optimizing enteral nutrition delivery by implementing volume-based feeding protocol for critically ill patients: an updated meta-analysis and systematic review
Source: Crit Care. 2023 May 5;27:173. doi: 10.1186/s13054-023-04439-0 (PMC10161662; doi:10.1186/s13054-023-04439-0)
Supplement: Supplementary file 2 — Additional file 2. Table S2. The results of quality assessment on Jadad for RCTs. [file 13054_2023_4439_MOESM2_ESM.docx]

TableS2 The results of quality assessment on modified Jadad Scores Scale for RCTs

| Study ID | Randomization | Concealment | Blinded | With or drop-out | Total |
| --- | --- | --- | --- | --- | --- |
| Kate Fetterplace, 2018 | 2 | 2 | 1 | 1 | 6 |
| Stephen A. McClave, 2014 | 2 | 2 | 0 | 1 | 5 |
| Yanxia Lu, 2020 | 2 | 1 | 0 | 1 | 4 |
| Guiyan Qi, 2020 | 2 | 1 | 2 | 0 | 5 |
| Daren K Heyland, 2013 | 2 | 1 | 1 | 1 | 5 |
| Shuangshuang Yang, 2022 | 1 | 1 | 1 | 0 | 3 |
