# Supplementary material for: Optimizing enteral nutrition delivery by implementing volume-based feeding protocol for critically ill patients: an updated meta-analysis and systematic review
Source: Crit Care. 2023 May 5;27:173. doi: 10.1186/s13054-023-04439-0 (PMC10161662; doi:10.1186/s13054-023-04439-0)
Supplement: Supplementary file 3 — Additional file 3. Table S3. The results of quality assessment on NOS for cohort studies. [file 13054_2023_4439_MOESM3_ESM.docx]

TableS3 The results of quality assessment on NOS for cohort studies

| Study ID | Selection | | | | Comparability | Outcome | | | Total |
| --- | --- | --- | --- | --- | --- | --- | --- | --- | --- |
| Elizabeth D. Krebs, 2018 | * | * | * | * | ** | * | * | * | ********* |
| Gaurav Sachdev, 2019 | * | * | * | * | ** | * |  |  | ******* |
| Ivy N. Haskins, 2015 | * | * | * | * | * | * |  |  | ****** |
| Susan Roberts, 2018 | * | * | * |  | * | * |  | * | ****** |
| JaNae Kinikin, 2019 | * | * | * | * | * | * |  | * | ******* |
| Amanda Holyk, 2019 | * | * | * |  | ** | * |  | * | ******* |
| Mina Bharal, 2019 | * | * | * | * | * | * |  | * | ******* |
| Phillip J. Prest, 2020 | * | * | * | * | * | * |  | * | ******* |
| Angela Bonomo, 2021 | * | * | * |  | ** | * |  | * | ******* |
| Jason McCartt, 2022 | * | * | * | * |  | * |  | * | ****** |
