# Supplementary material for: Optimizing enteral nutrition delivery by implementing volume-based feeding protocol for critically ill patients: an updated meta-analysis and systematic review
Source: Crit Care. 2023 May 5;27:173. doi: 10.1186/s13054-023-04439-0 (PMC10161662; doi:10.1186/s13054-023-04439-0)
Supplement: Supplementary file 4 — Additional file 4. Table S4. The results of quality assessment on ROB2 for RCT. [file 13054_2023_4439_MOESM4_ESM.docx]

TableS4 The results of quality assessment on ROB2 for RCT

| ***Study ID*** | ***Experimental*** | ***Comparator*** | ***Outcome*** | ***Weight*** | ***D1*** | ***D2*** | ***D3*** | ***D4*** | ***D5*** | ***Overall*** |  |  |  |
| --- | --- | --- | --- | --- | --- | --- | --- | --- | --- | --- | --- | --- | --- |
| *Kate Feteerplace*  *2018* | *NA* | *NA* | *NA* | *1* | *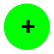* | *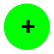* | *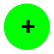* | *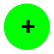* | *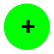* | *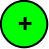* |  | *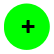* | *Low risk* |
| *Stephen A. McClave*  *2014* | *NA* | *NA* | *NA* | *1* | *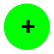* | *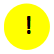* | *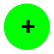* | *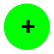* | *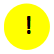* | *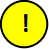* |  | *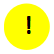* | *Some concerns* |
| *Yanxia Lu*  *2020* | *NA* | *NA* | *NA* | *1* | *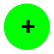* | *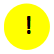* | *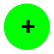* | *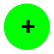* | *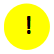* | *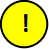* |  | *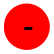* | *High risk* |
| *Guiyan Qi*  *2020* | *NA* | *NA* | *NA* | *1* | *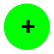* | *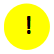* | *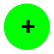* | *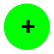* | *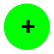* | *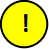* |  | *D1*  *Randomisation process*  *D2*  *Deviations from the intended interventions*  *D3*  *Missing outcome data*  *D4*  *Measurement of the outcome*  *D5*  *Selection of the reported result* | |
| *Daren K Heyland*  *2015* | *NA* | *NA* | *NA* | *1* | *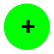* | *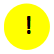* | *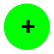* | *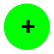* | *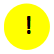* | *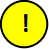* |  |  |  |
| *Shuangshuang Yang*  *2022* | *NA* | *NA* | *NA* | *1* | *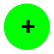* | *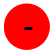* | *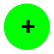* | *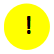* | *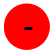* | *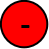* |  |  |  |
|  | | | | | | | | | | |  |  |  |
|  |  |  |  |  |  |  |  |  |  |  |  |  |  |
|  |  |  |  |  |  |  |  |  |  |  |  |  |  |
