# Supplementary figures and images for: Optimizing enteral nutrition delivery by implementing volume-based feeding protocol for critically ill patients: an updated meta-analysis and systematic review
Source: Crit Care. 2023 May 5;27:173. doi: 10.1186/s13054-023-04439-0 (PMC10161662; doi:10.1186/s13054-023-04439-0)

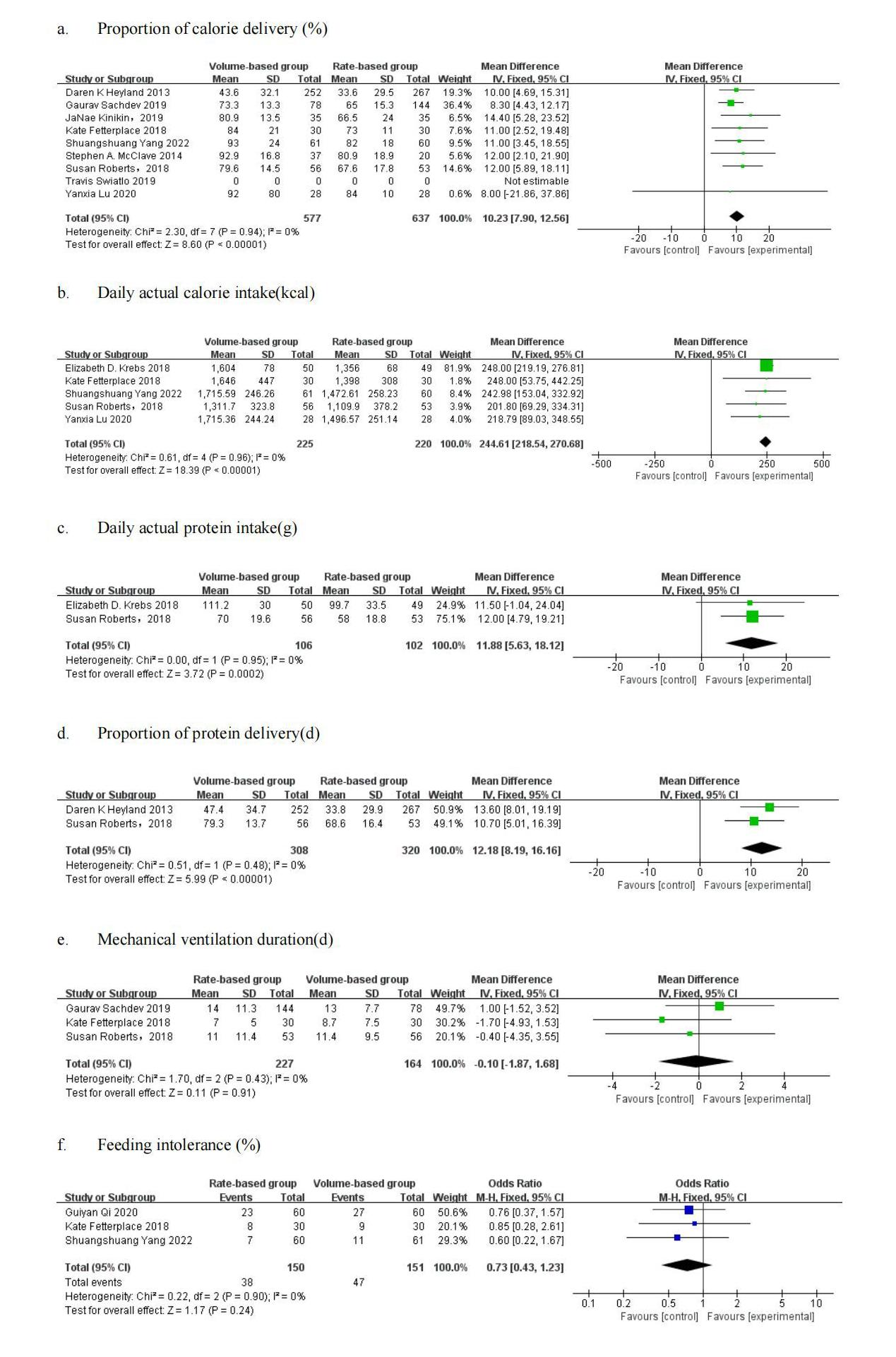

Supplement: Supplementary file 5 — Additional file 5. Figure S1. The results of sensitivity analysis. [file 13054_2023_4439_MOESM5_ESM.jpg]
