# Supplementary material for: Optimizing enteral nutrition delivery by implementing volume-based feeding protocol for critically ill patients: an updated meta-analysis and systematic review
Source: Crit Care. 2023 May 5;27:173. doi: 10.1186/s13054-023-04439-0 (PMC10161662; doi:10.1186/s13054-023-04439-0)
Supplement: Supplementary file 6 — Additional file 6. Table S5. The results of Grade of Recommendations Assessment, Development and Evaluation. [file 13054_2023_4439_MOESM6_ESM.docx]

TableS5 The results of Grade of Recommendations Assessment, Development and Evaluation(GRADE)

| **Certainty assessment** | | | | | | | **№ of patients** | | **Effect** | | **Certainty** |
| --- | --- | --- | --- | --- | --- | --- | --- | --- | --- | --- | --- |
| **№ of studies** | **Study design** | **Risk of bias** | **Inconsistency** | **Indirectness** | **Imprecision** | **Other considerations** | **VBF group** | **RBF group** | **Relative (95% CI)** | **Absolute (95% CI)** |  |
| **Daily calorie received (g)** | | | | | | | | | | | |
| 7 | RCT and cohort study | not serious | not serious | not serious | not serious | none | 575 | 444 | - | MD 386.61 higher | ⨁⨁⨁⨁ |
|  |  |  |  |  |  |  |  |  |  | (180.32 higher to 592.91 higher) | High |
| **Percentage of goal energy received (%)** | | | | | | | | | | | |
| 11 | RCT and cohort study | not serious | not serious | not serious | not serious | none | 769 | 930 | - | MD 15.41 higher | ⨁⨁⨁⨁ |
|  |  |  |  |  |  |  |  |  |  | (10.68 higher to 20.14 higher) | High |
| **Completion of 80% energy delivery (n/N)** | | | | | | | | | | | |
| 3 | Cohort study | not serious | not serious | not serious | not serious | none | 255/399 (63.9%) | 188/500 (37.6%) | **OR 2.84** | **255 more per 1,000** | ⨁⨁◯◯ |
|  |  |  |  |  |  |  |  |  | (2.13 to 3.78) | (from 186 more to 319 more) | Low |
| **Daily protein received (g)** | | | | | | | | | | | |
| 5 | RCT and cohort study | not serious | not serious | not serious | not serious | none | 486 | 356 | - | MD 31.44 higher | ⨁⨁⨁⨁ |
|  |  |  |  |  |  |  |  |  |  | (9.48 higher to 53.4 higher) | High |
| **Proportion of protein delivery (g)** | | | | | | | | | | | |
| 4 | RCT and cohort study | not serious | not serious | not serious | not serious | none | 393 | 377 | - | MD 22.05 higher | ⨁⨁⨁⨁ |
|  |  |  |  |  |  |  |  |  |  | (10.89 higher to 33.22 higher) | High |
| **Mortality (n/N)** | | | | | | | | | | | |
| 10 | RCT and cohort study | not serious | seriousa | not serious | not serious | none | 160/793 (20.2%) | 201/931 (21.6%) | **RR 0.97** | **6 fewer per 1,000** | ⨁⨁⨁◯ |
|  |  |  |  |  |  |  |  |  | (0.80 to 1.17) | (from 43 fewer to 37 more) | Moderate |
| **Median ICU length of stay(d)** | | | | | | | | | | | |
| 4 | RCT and cohort study | not serious | seriousa | not serious | seriousc | none | 224 | 287 | - | MD 0.78 lower | ⨁⨁◯◯ |
|  |  |  |  |  |  |  |  |  |  | (1.56 lower to 0.01 lower) | Low |
| **Median mechanical ventilation duration(d)** | | | | | | | | | | | |
| 4 | RCT and cohort study | not serious | seriousd | not serious | seriousc | none | 224 | 287 | - | MD 0.81 lower | ⨁⨁◯◯ |
|  |  |  |  |  |  |  |  |  |  | (1.92 lower to 0.3 higher) | Low |
| **Emesis(n/N)** | | | | | | | | | | | |
| 5 | RCT and cohort study | not serious | seriousa | not serious | seriousb | none | 26/294 (8.8%) | 39/487 (8.0%) | **RR 0.82** | **14 fewer per 1,000** | ⨁⨁◯◯ |

**CI:** confidence interval; **MD:** mean difference; **OR:** odds ratio; **RR:** risk ratio

#### Explanations

a. At least one study reports contrary result.

b. The range of 95%CI is wide.

c. The number of patients are not enough.

d. There are almost half of studies with contrary result.
